# Can markers of biological age predict dependency in old age?

**DOI:** 10.1007/s10522-019-09795-5

**Published:** 2019-01-21

**Authors:** Juulia Jylhävä, Miao Jiang, Andrea D. Foebel, Nancy L. Pedersen, Sara Hägg

**Affiliations:** The Department of Medical Epidemiology and Biostatistics, Karolinska Institutet, Nobels väg 12A, 17 156 Stockholm, Sweden

**Keywords:** Epigenetic clock, Leukocyte telomere length, Frailty index, Need for care, ADL

## Abstract

Recent research has shown that markers of biological age, such as leukocyte telomere length (LTL), epigenetic clocks and the frailty index (FI) are predictive of mortality and age-related diseases. However, whether these markers associate with the need for care in old age, thereby having utility in reflecting dependency, is unclear. This study was undertaken to analyze whether LTL, two epigenetic clocks—the DNA methylation age (DNAmAge) and DNAm PhenoAge—and the FI are associated with the need for regular care in up to 604 individuals (aged 48–94 years) participating in the Swedish Adoption/Twin Study of Aging. Need for regular care was defined as receiving formal or informal help in daily routines at least once per week. Logistic regression adjusted for age, sex and education was used in the analysis. The predictive accuracies, assessed as the area under the curve (AUC) for the significant biological age measures were further compared to the accuracies of the limitations in activities of daily living (ADL) and instrumental ADL (IADL). Neither LTL nor the epigenetic clocks were associated with the need for care, whereas the FI was; odds ratio for 10% increase in FI 3.54 (95% confidence interval 2.32–5.41). The FI also demonstrated higher predictive accuracy than the ADL score (FI AUC 0.80 vs. ADL score AUC 0.62; p < 0.001 for equality of the AUCs), whereas the difference between FI AUC (0.80) and IADL score AUC (0.75) was not significant (p = 0.238). The FI might thus be a useful marker for the need for care.

**Electronic supplementary material:**

The online version of this article (10.1007/s10522-019-09795-5) contains supplementary material, which is available to authorized users.

## Introduction

A central feature of aging is loss of function and independence in performing daily routines. Dependency, or needing help in daily activities, has a wide range of consequences both on the individual and society. Having limitations in activities of daily living (ADL) increases the risk of institutionalization (Gaugler et al. 2007) and is associated with decreased quality of life (Baernholdt et al. 2012). On a societal level, transition to dependency at home or moving to a nursing home is accompanied by a substantial increase in health care costs (Fried et al. 2001; Guralnik et al. 2002). Due to the major demographic shift towards the growth of aging populations and the fact that most older adults will receive care at some point at the end of life (Bravell et al. 2008; Kingston et al. 2017), it is pertinent to identify markers for dependency. Such markers would ideally catch individuals at lower levels of dependency and have utility in interventions aimed at decreasing the number of remaining years of life spent with high dependency—i.e., extending healthspan.

Several markers of biological age, such as leukocyte telomere length (LTL), the frailty index (FI) and composite markers of DNA methylation sites, termed epigenetic clocks, have demonstrated utility in predicting mortality and other age-related outcomes independent of chronological age and other risk factors (Chen et al. 2016; Horvath and Raj 2018; Jylhava et al. 2017; Vermeiren 2016). These biological age markers have also been associated with clinical biomarkers and functional performance measures (Horvath and Raj 2018; Hubbard et al. 2009; Jylhava et al. 2017; Levine et al. 2018). In this regard, these biological age markers hold great promise as indicators of how fit individuals are for their age and provide information above and beyond chronological age. However, clinical indicators, such as the FI and cellular markers, such as the LTL and epigenetic clocks that do not require clinical variables in the assessment, likely reflect different aspects of physiological and biological aging. Comparative analyses on these markers are thus needed to establish their relationships with different aging phenotypes and healthspan.

However, whether the markers of biological age associate with the need for care in old age and could thereby have utility in predicting dependency is unknown. The necessity for finding sensitive measures of the need for care was recently highlighted by a survey that concluded that half of older people with social care needs have unmet needs for at least some of their difficulties (Ipsos MORI 2017). In Sweden, as well as in many countries worldwide, administration of social care is largely based on ADL. Self-reported ADLs are, however, only modestly related to actual performance of different motor functions (Bravell et al. 2011). To this end, this study was undertaken to analyze if LTL, FI (Mitnitski et al. 2001; Searle et al. 2008) and two epigenetic clocks, the DNA methylation age (DNAmAge) (Horvath 2013) and DNAm phenotypic age (DNAm PhenoAge) (Levine et al. 2018) were associated with the need for regular care i.e., assistance in daily routines at least once per week. To further explore their relationship with the need for care, we sought to analyze if those markers that demonstrated a significant association with the need for care, were also associated with the amount of care needed per week. Lastly, we sought to evaluate the predictive accuracies of the significant markers and compare with the predictive accuracies of the ADL and instrumental ADL (IADL) scores—two scales that are commonly used in assessing the need of formal social care.

## Methods

### Study cohort

The Swedish Adoption/Twin Study of Aging (SATSA) is a longitudinal population-based cohort of same-sex twin pairs reared together and reared apart (Finkel and Pedersen 2004) drawn from the Swedish Twin Registry (Magnusson et al. 2013). SATSA was initiated in 1984 and since then, a total of nine questionnaire waves and ten in-person testing (IPT) waves have been performed. The timeline of the SATSA data collection, sampling procedures and the data sets through the seventh questionnaire and seventh IPT wave have been previously described (Pedersen 2015). The sample used in this study consisted of individuals participating at IPTs 3, 5 and 6, who had data available on LTL, epigenetic clocks and FI. For each individual, the first IPT wave when all the biological age markers and the outcome were available was used in constructing the cross-sectional sample for the present study. The total sample consisted of 687 individuals (281 men, 406 women; aged 48-94 years) of whom 576 had LTL data, 361 had epigenetic clock data and 604 had FI data available. The overlap in the availabilities of the biological age markers is presented in Online Resource 1. Due to the incomplete overlap, separate models were fitted for each of the markers to maximize power (see “Methods” section).

### Study variables

LTL was measured from whole blood leukocytes using a qPCR assay that yielded a ratio of a telomere length PCR product (T) to a PCR product of a reference gene (S), denoted as the T/S-ratio. Detailed methodology for the SATSA LTL assessment has been previously described (Berglund et al. 2016).

Whole blood methylation data were obtained using the Infinium 450 K HumanMethylation BeadChip (Illumina, San Diego, CA, USA). Processing of the samples has been previously described (Wang et al. 2018). The DNAmAge is a composite measure of 353 CpG sites that best predicted chronological age (Horvath 2013). The DNAm PhenoAge is a composite measure that consists of 513 CpG sites that regressed against chronological age and nine markers of phenotypic aging: albumin, creatinine, glucose, C-reactive protein, lymphocyte percentage, mean cell volume, red blood cell distribution width, alkaline phosphatase and white blood cell count (Levine et al. 2018). Hence, by definition the DNAm PhenoAge taps into physiological dysregulation, whereas the DNAmAge builds exclusively upon the information on the association between chronological age and DNA methylation. The DNAmAge was assessed using the algorithm described by Horvath (Horvath 2013) (available at https://dnamage.genetics.ucla.edu/home). The algorithm for the DNAm PhenoAge was obtained from Levine et al. by request. Normalized data (beta values) were used in the algorithms to derive the DNAmAges, and the values were further adjusted for blood cell distributions and chronological age to yield epigenetic clock residuals. These residuals, hereafter denoted as the DNAmAge and DNAm PhenoAge residuals, were used in the models. The blood cell type distributions (monocytes, granulocytes, B cells, CD4 and CD8 and NK cells) for both clocks were assessed in the Horvath online calculator (https://dnamage.genetics.ucla.edu/home) using the Houseman method (Houseman et al. 2012). Construction and validation of an FI, also called a deficit accumulation model, for the second SATSA questionnaire wave in 1987 has been previously described (Jiang et al. 2017). The FIs for IPTs 3, 5 and 6 used in this study were constructed identically to the 1987 questionnaire wave. Briefly, it includes 42 self-reported health deficits that cover a wide range of bodily systems (Online Resource 2) and thus represents a multidimensional approach to measuring frailty. All the included FI items meet the standard inclusion criteria (Searle et al. 2008). The FI was assessed by counting the number of deficits and dividing the count by the total number of deficits considered. For example, an individual having 10/42 deficits has an FI of 0.238. Although the theoretical maximum of the FI is 1, in almost every cohort, > 99% of individuals have an FI < 0.7, indicating that accumulation of deficits beyond this point is lethal (Rockwood and Mitnitski 2006). For logistic regression, the FI was multiplied by ten so that the odds ratio (OR) is interpretable as the risk associated with 10% increase in the FI. Education was categorized into four levels: 1 = primary education, 2 = lower secondary or vocational, 3 = upper secondary education, and 4 = tertiary education. Limitations in ADL were assessed using a modified Katz scale for which the following five activities were considered: toileting, dressing and undressing, getting in and out of bed, showering/bathing and eating. Each activity item was assigned a score of 0 = independent, 1 = needs help and 2 = cannot perform the activity, yielding a total score ranging from 0 to 10. Limitations in seven IADLs with the corresponding scoring were assessed on the Lawton’s scale: ability to use telephone, (0 = can look up numbers, dial or answer; 1 = doesn’t use phone), grocery shopping (0 = can shop; 1 = needs help or doesn’t shop), food preparation (0 = can plan and prepare; 1 = can only heat up or doesn’t cook), housework (0 = can perform with or without help; 1 = cannot perform), travelling/transportation (0 = can travel alone or go by taxi; 1 = needs helper, special assistance or doesn’t travel), management of medications (0 = can manage; 1 = needs help or cannot manage) and management of finances (0 = can manage with or without help; 1 = cannot manage). Scores across the items were summed, yielding a total score ranging from 0 to 7. The need for any kind of social care, formal or informal, was assessed identically in all questionnaire waves by asking “Do you regularly, at least once a week, receive help or are looked after”? “No” was coded as 0 and “Yes” was coded as 1, and this variable was used as the outcome in the logistic regression for the need for care. For individuals responding’yes’ to the need for care, the following question was used to assess the amount of care needed: “How many days per week do you receive help or get looked after by e.g. an immediate family member, relatives, social worker, health staff or similar”? Based on this information, the individuals were grouped into three classes: (i) those not reporting needing care, (ii) those reporting needing care once or twice a week and iii) those needing care ≥ 3 times a week. This 3-level categorization was not used in the logistic regression; instead, it was used to analyze if those markers that show an association with the need for care in the logistic regression also show an association with the amount of care needed.

### Statistical analyses

Logistic regression was used to assess the associations between the markers of biological age (LTL, the DNAmAge and DNAm PhenoAge residuals and the FI) and the need for care. Not needing care was used as the reference category. Due to the incomplete overlap in the markers (Online Resource 1), separate models were fitted for each of the markers to maximize power. The models were adjusted for age, sex (men as reference) and education level (treated as an ordinal variable). However, as the epigenetic clock measures were residuals already adjusted for age (and cell counts), only sex and education were entered as covariates. Clustering of the data in twin pairs was accounted for in the modeling by using clustered robust standard errors for the coefficients.

To analyze the association between the amount of care needed (i.e., the 3-level categories) and the markers demonstrating an association in the logistic model, the Mann–Whitney U test for differences in ranked distributions was used. Comparisons were performed pairwise, contrasting category 1 (no need for help) to category 2 (needing help once or twice a week) and category 2 to category 3 (needing help three or more times a week).

The predictive accuracy of the biological age markers that were significantly associated with the need for care in the logistic model was compared to that of the ADL and IADL scores. The predictive accuracies were assessed using the area under the receiver operating characteristic (AUC/ROC) curves, and the equality of the AUCs were assessed using the roccomp function in Stata. As our 42-item FI contained items of ADL and IADL (indicated in the Online Resource 2), we constructed a 29-item FI that was stripped of the 13 ADL and IADL items. The procedure for creating the 29-item FI was identical to that for the 42-item FI (Jiang et al. 2017) but without inclusion of the 13 ADL and IADL items (Online Resource 2). The 29-item FI was used for a sensitivity analysis in logistic regression for the need for care (age, sex and education included as covariates) and for comparison of the predictive accuracies between the FI and ADL and IADL; this way we were able to make unbiased comparisons between the predictive accuracies of the FI, ADL and IADL. In all analyses, p-values < 0.05 were considered statistically significant. Statistical analyses were performed using Stata version 14.1 (College Station, TX: StataCorp LP).

## Results

Characteristics of the study population are presented in Table 1. As the epigenetic clock data were available for a smaller sample than the FI and LTL, the left hand side of Table 1 presents the sample characteristics separately for those with the epigenetic clocks available. The number of individuals needing care in the logistic regression was 51/576 for LTL model, 29/361 for both of the epigenetic clocks’ models and 57/604 for the FI model. Neither the LTL nor the epigenetic clocks were associated with the need for care, but a significant association independent of age, sex and education was observed between the FI (42-item) and the need for care (Table 2). A 10% increase in the FI was associated with 3.54-fold increased odds for needing care. Higher age was also associated with increased odds of needing care (odds ratio [OR] for 1 year increase in age 1.15, 95% confidence interval [CI] 1.10–1.22 in the model for the FI). Sex and education level were not associated with the need for care (Table 2). Sensitivity analysis using the 29-item FI in the logistic regression for the need for care revealed that the FI, also when constructed without ADL and IADL items, is associated with the need for care, independent of age, sex and education (HR for 10% increase in the 29-item FI 2.02, 95% CI 1.54–2.67, p > 0.001).

**Table 1:** Characteristics of the study population

	Overall SATSA sample (n = 361–687)	Epigenetic clock data available (n = 361)
Age (years)	69.23 (9.66)	68.59 (9.65)
Women^a^	406 (59.1)	218 (60.4)
Education^a^		
Primary education	389 (56.6)	203 (56.2)
Lower secondary or vocational	197 (28.7)	99 (27.4)
Upper secondary education	51 (7.4)	28 (7.8)
Tertiary education	50 (7.3)	31 (8.6)
FI (42-item)^b^	0.08 (0.10)	0.08 (0.11)
LTL	0.75 (0.24)	0.76 (0.26)
DNAmAge	45.27 (8.60)	45.27 (8.60)
DNAmAge residual*	− 0.35 (6.38)	− 0.35 (6.38)
DNAm PhenoAge	65.64 (10.13)	65.64 (10.13)
DNAm PhenoAge residual*	− 0.48 (7.22)	− 0.48 (7.22)
ADL score^c^	83 (12.8)	47 (13.8)
IADL score^c^	65 (10.4)	36 (9.9)
Need for care^a^		
Yes	66 (9.6)	29 (8.0)
No	621 (90.4)	332 (92.0)
Amount of care received^a^		
0 times a week	621 (91.5)	332 (92.0)
1–2 times a week	22 (3.2)	11 (3.1)
≥ 3 times a week	36 (5.5)	15 (4.2)

Mean and standard deviation is presented unless otherwise indicated. Overall sample n **= **687, n = 604 for FI, n = 576 for LTL, n = 361 for the DNAmAge and DNAm PhenoAge and n = 658 for the ADL score, n = 625 for the IADL score and n = 679 for the amount of care needed

*ADL* activities of daily living, *DNAmAge* DNA methylation age, *FI* frailty index, *IADL* instrumental activities of daily living, *IQR* interquartile range, *LTL* leukocyte telomere length

^a^Values are n(%)

^b^Values are median (interquartile range)

^c^Values are n(%) for individuals with ADL score ≥ 2 and IADL score ≥ 1

*The epigenetic clock residuals are derived from the corresponding DNAmAge measure by regressing out the effect of age and blood cell proportions

**Table 2:** Logistic regression models for the biological age markers and the need for care

Model	OR	95% CI	p
LTL			
Age	1.20	1.14–1.27	< 0.001
Sex	0.73	0.34–1.54	0.403
Education	0.59	0.33–1.06	0.076
LTL	1.26	0.49–3.24	0.626
DNAmAge residual			
Age	1.21	1.14–1.28	< 0.001
Sex	0.97	0.33–2.83	0.958
Education	0.41	0.14–1.21	0.107
DNAmAge residual	0.96	0.90–1.04	0.330
DNAm PhenoAge residual			
Age	1.20	1.13–1.28	< 0.001
Sex	1.07	0.39–2.97	0.892
Education	0.42	0.15–1.18	0.099
DNAm PhenoAge residual	0.99	0.93–1.05	0.668
FI			
Age	1.15	1.10–1.22	< 0.001
Sex	0.59	0.30–1.18	0.139
Education	0.93	0.57–1.53	0.782
FI	3.54	2.32–5.41	< 0.001

Separate models were fitted for each of the markers

*CI* confidence interval, *FI* frailty index, *LTL* leukocyte telomere length, *OR* odds ratio

**Fig. 1:**
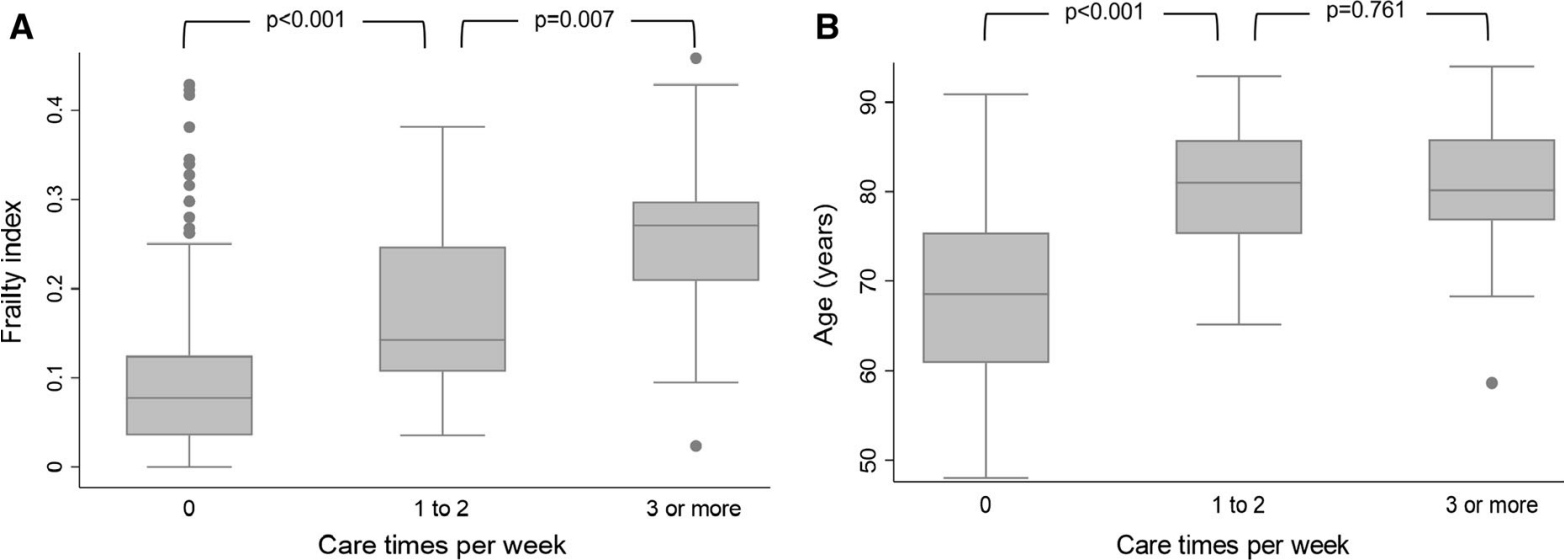
Distributions of the frailty index (**a**) and age (**b**) according by the amount of care needed. For the frailty index, differences in distributions were significant across all groups. For age, the difference was significant only between those not needing care at all and those needing care once or twice per week

The amount of care needed, assessed as days per week, was associated with the (42-item) FI level (Mann–Whitney U test p < 0.05 for pairwise comparisons). The highest FI levels were observed among those who needed care 3 or more times a week (Fig. 1a). A significant association between age and the amount of care needed was observed only between those not needing care at all and those needing care once or twice per week (Fig. 1b). No difference in age was observed between those needing care once or twice per week and those needing care 3 times or more per week (Fig. 1b).

**Fig. 2:**
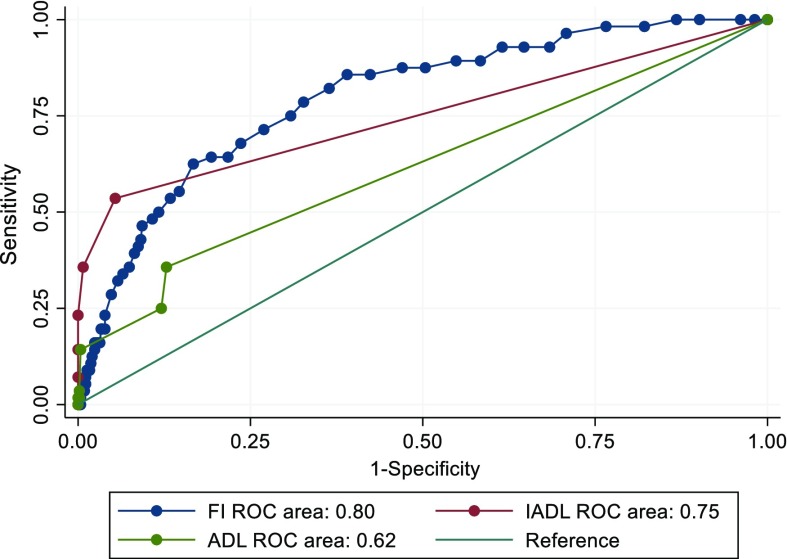
Predictive accuracies for the 29-item FI, the ADL and IADL scores for predicting dependency. Test for the equalities between the FI and ADL score AUC/ROCs indicated that the FI demonstrated significantly better predictive accuracy (p < 0.001) whereas the difference between the FI and IADL score AUC/ROCs was not statistically significant (p = 0.238). Abbreviations: ADL, activities of daily living; AUC, area under the curve; IADL, instrumental activities of daily living; FI, frailty index; ROC, receiver operating characteristic

Comparison of the (29-item) FI and ADL score AUCs demonstrated that the FI had significantly better overall predictive accuracy for the need for care (AUC/ROC 0.80, 95% CI 0.74–0.86 for FI vs. AUC/ROC 0.62, 95% CI 0.55–0.68 for ADL score; p < 0.001 for equality of the AUC/ROC curves; Fig. 2). Although the AUC/ROC estimate was higher for the FI than for IADL score, the difference was not statistically significant (AUC/ROC FI 0.80, 95% CI 0.74–0.86 vs. AUC/ROC IADL score 0.75, 95% CI 0.68–0.82; p < 0.238; Fig. 2). The FI values nevertheless tended to exhibit higher sensitivity (true positive rate) than ADL and IADL scores yet the FI values also exhibited higher false positive rates (1-specificity) than ADL and IADL scores (Fig. 2).

## Discussion

In this study, we analyzed if four common markers of biological age–LTL, the DNAmAge and DNAm PhenoAge residuals and the FI–associate with dependency, measured as the need for regular care, in a Swedish population-based cohort. We found that only the FI was associated with the need for care, independent of age, sex and education level. A 10% increase in the FI was associated with a more than threefold increased odds for needing care. Higher age was also a risk factor for needing care. When the amount of care needed (times per week) was categorized into three categories, we found an increase in the level of frailty with an increase in the amount of care needed across all three categories. A similar “dose–response” pattern was not found between age and the amount of care needed as the difference in age was significant only between the first two categories. Comparison of the predictive accuracies of the FI and the ADL and IADL scores revealed that the FI had significantly greater overall accuracy than the ADL score but not significantly greater than the IADL score. The FI values nevertheless demonstrated higher sensitivity rates than ADL and IADL scores but were also more prone to false positives.

As the need for regular care in our study was assessed as receiving any formal or informal assistance in daily routines at least once per week, it represents a form of social care provided at the residence of the individual. By definition, it is somewhat distinct from ADL and IADL limitations and dependency on medical care and thus taps into dependency from a different angle. This type of dependency has rarely been analyzed in the context of finding markers and determinants for it. Jotheeswaran et al. (2015) used a series of open-ended questions to assess the need for care inside and outside home, and showed in a large population-based study involving Latin America, India and China that two different phenotypic measures of frailty and their aggregate predicted the onset of dependency during a median follow-up of 3.9 years (Jotheeswaran et al. 2015). The association remained significant after adjusting for multimorbidity and disability. Although they did not use the FI to measure frailty, but instead assessed frailty based on exhaustion, weight loss, slow walking speed, physical inactivity, undernutrition, and cognitive and sensory impairment, our results are consistent with their finding that the level of frailty is associated with dependency. As our results further demonstrated that the FI performs better than ADL in identifying those in the need of regular care, we propose that frailty assessment could be further studied as an additional measure in weighing the need of formal (social) care. However, as needing social care and ADL/IADL are by definition correlated, more research should also be done in resolving those potentially unidentified aspects of dependency that the FI covers but the (I)ADLs do not. The ADL and IADL scores indeed exhibited low false positive rates for the need for care whereas the FI reached higher sensitivity rates. Furthermore, more comparative studies in larger populations that present higher levels of dependency and disability are warranted, as our sample was rather small and well-functioning in terms of ADL and IADL limitations and the level of frailty. A larger sample is also required to establish a firm dose–response relationship between the FI and the amount of care needed as we did not have enough power to model the association.

Although neither the LTL nor the epigenetic clocks were associated with the need for care in our study, these markers have previously been associated with related phenotypes, such as disability and physical functioning. A U.S.-based study demonstrated that individuals with shorter LTL were more likely to have ADL and IADL disabilities (Risques et al. 2010) and a multi-center European study found that individuals in the lowest quintile of LTL had a higher likelihood of having physical limitation compared to those in other quintiles (Montiel Rojas et al. 2018). Higher DNAm PhenoAge values have been associated with an increase in physical functioning problems (Levine et al. 2018) and higher DNAmAge values have demonstrated cross-sectional correlations with poorer fitness, measured as grip strength and lung function (Marioni et al. 2015). However, as the epigenetic clocks are rather newly introduced markers, further research is needed to analyze their associations with dependency outcomes.

Nursing home admission, usually characterized by a medium to high level dependency, is a frequently analyzed dependency outcome. A myriad of predictors for nursing home placement have been identified, ADL limitations and cognitive decline being among the foremost (Puts et al. 2017). The level of frailty, measured using various frailty scales, also has predictive ability for nursing home placement (Kojima 2018; Vermeiren S 2016). However, in societies such as Sweden that are increasingly encouraging older individuals to live in their own homes instead of nursing homes (“stay-in-place-policy”)(The Swedish Institute 2013), it may be more pertinent to focus on identifying the determinants of care needs regardless of the form of housing. Effective identification of low dependency could increase the likelihood of interventions succeeding in extending the healthspan, and also less intensive interventions would make an impact. Along these lines, a recommendation to target frailty rather than disability has been put forward as disability is difficult to reverse (European Comission Action Group A3 2016). In this respect, frailty may provide a more amenable target due to its responsiveness to intervention (Puts et al. 2017). Interventions based on physical exercise have been advocated as the means to reverse frailty and decrease the risk of nursing home placement (Kojima 2018). However, as there are several scales to measure frailty, a consensus on how to measure it best in health care settings needs to be reached (Morley et al. 2013).

In conclusion, we find that biological age, measured as the FI, is independently associated with the need of regular social care, and thus represents a stronger candidate marker for dependency than the LTL and epigenetic clocks. In this respect, the analyzed markers of biological age appear to capture different aspects of fitness in aging. This finding is however not surprising, as the FI is a multidimensional measure, covering various domains of health and associated with a host of negative aging outcomes (Vermeiren 2016). We also found that the FI outperforms ADL in overall predictive accuracy, and could thus be further studied for its usefulness to inform social care administration.

## Data availability

SATSA methylation data are available in the ArrayExpress database at EMBL-EBI (www.ebi.ac.uk/arrayexpress) under accession number E-MTAB-7309.

## Electronic supplementary material

Below is the link to the electronic supplementary material.

Supplementary material 1 (PDF 202 kb)

Supplementary material 2 (PDF 271 kb)
