# Supplementary material for: Can markers of biological age predict dependency in old age?
Source: Biogerontology. 2019 Jan 21;20(3):321–9. doi: 10.1007/s10522-019-09795-5 (PMC6535415; doi:10.1007/s10522-019-09795-5)
Supplement: Supplementary file 1 — Supplementary material 1 (PDF 202 kb) [file 10522_2019_9795_MOESM1_ESM.pdf]

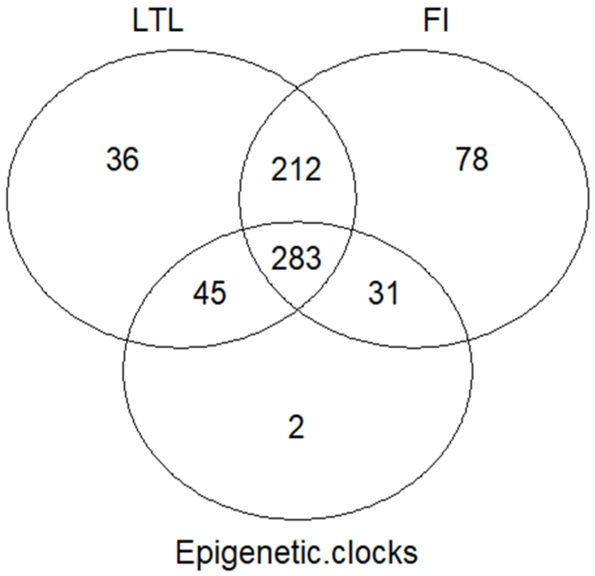

Online Resource 1. A Venn diagram demonstrating the overlap in the availability of the different markers of biological age. Abbreviations: LTL, leukocyte telomere length, FI, frailty index.
