# Supplementary material for: Can markers of biological age predict dependency in old age?
Source: Biogerontology. 2019 Jan 21;20(3):321–9. doi: 10.1007/s10522-019-09795-5 (PMC6535415; doi:10.1007/s10522-019-09795-5)
Supplement: Supplementary file 2 — Supplementary material 2 (PDF 271 kb) [file 10522_2019_9795_MOESM2_ESM.pdf]

**Online Resource 2.** List of the 42 items included in the frailty index and their scoring. For creating the 29-item FI, the 13 items of ADL and IADL were removed from the 42-item FI.

| **Item** | **Scoring** |
|---|---|
| Hearing status | Perfect=0, Good=0.25, Pretty Good=0.5, Bad=0.75, Deaf or almost deaf=1 |
| Vision status | Perfect=0, Good=0.25, Pretty Good=0.5 Bad=0.75, Blind or almost blind=1 |
| Health prevents from doing things normally would like to do | No=0, Somewhat=0.5, Yes=1 |
| Self-reported general health | Good=0, Mediocre=0.5, Bad=1 |
| Cancer or leukemia | No=0, Yes=1 |
| Rheumatoid arthritis | No=0, Yes=1 |
| Arthritis | No=0, Yes=1 |
| Chronic bronchitis or emphysema | No=0, Yes=1 |
| Cataracts | No=0, Yes=1 |
| Chest pain | No=0, Yes=1 |
| Circulation problems in arms or legs | No=0, Yes=1 |
| Persistent cough | No=0, Yes=1 |
| Diabetes | No=0, Yes=1 |
| Goiter or other gland problems | No=0, Yes=1 |
| Heart failure | No=0, Yes=1 |
| Hypertension | No=0, Yes=1 |
| Kidney disease | No=0, Yes=1 |
| Brittle bones | No=0, Yes=1 |
| Sciatica | No=0, Yes=1 |
| Anemia | No=0, Yes=1 |
| Cerebral hemorrhage or blood clot in brain | No=0, Yes=1 |
| Dizziness | No=0, Yes=1 |
| Gastric ulcer | No=0, Yes=1 |
| Allergies/allergic manifestations | No=0, Yes=1 |
| Asthma | No=0, Yes=1 |
| Shower and bathe[1] | No problem=0, Needs help=0.5, Cannot=1 |
| Get in and out of bed[1] | No problem=0, Needs help=0.5, Cannot=1 |
| Dress and undress[1] | No problem=0, Needs help=0.5, Cannot=1 |
| Self-grooming[1] | No problem=0, Needs help=0.5, Cannot=1 |
| Walking[1] | No problem=0, Needs help=0.5, Cannot=1 |
| Trouble getting to toilet in time[1] | No=0, Yes=1 |
| Travel further distances[2] | Can travel alone=0, Can go by taxi=0.5, Needs helper, special assistance or doesn't travel=1 |
| Housework[2] | No problems=0, Needs help=0.5, Doesn't do=1 |
| Prepare meals[2] | Can plan/prepare=0, Can heat up=0.5, Doesn't cook=1 |
| Manage medications[2] | No problems=0, Needs help=0.5, Doesn't do=1 |
| Manage money[2] | No problems=0, Needs help=0.5, Doesn't do=1 |
| Use telephone[2] | Can look up numbers and dial=0 Needs help or doesn't use phone=1 |
| Grocery shopping[2] | Can shop=0, Needs help=0.5, Doesn't shop=1 |

| | |
|---|---|
| Feeling lonely[3] | Never, almost never, rather seldom=0<br>Quite often, always, almost always=1 |
| Feeling depressed[3] | Never, almost never or rather seldom=0<br>Quite often, always, almost always=1 |
| Consider oneself happy and carefree | No=1, Yes=0 |
| Usually feels tired | No=0, Yes=1 |

Note. [1]from the instrument of basic Activities of Daily Living (ADL), [2]from the instrument of Instrumental Activities of Daily Living (IADL), [3] from the Center for Epidemiologic Studies Depression Scale.
